# Sodium-glucose cotransporter-2 inhibitors and the risk for liver abscess in patients with type 2 diabetes mellitus: a nationwide retrospective cohort study

**DOI:** 10.1038/s41598-026-50136-7

**Published:** 2026-05-02

**Authors:** Wu-Lung Chuang, Ruey-Hwang Chou, Cheng-Li Lin, Der-Yang Cho, Kuang-Hsi Chang

**Affiliations:** 1 Division of Endocrinology and Metabolism, Department of Internal Medicine, Changhua Christian Hospital, Changhua, 500 Taiwan; 2 Division of Endocrinology and Metabolism, Department of Internal Medicine, Lukang Christian Hospital, Changhua, 505 Taiwan; 3 Graduate Institute of Biomedical Sciences, China Medical University, Taichung, 40447 Taiwan; 4 Center for Molecular Medicine, China Medical University Hospital, Taichung, 404 Taiwan; 5 Department of Medical Laboratory and Biotechnology, Asia University, Taichung, 413 Taiwan; 6 Management Office for Health Data, China Medical University Hospital, Taichung, 404 Taiwan; 7 College of Medicine, China Medical University, Taichung, 404 Taiwan; 8 Translational Cell Therapy Center, Department of Medical Research, Medical University Hospital, Taichung, Taiwan 40447 China; 9 Department of Neurosurgery, China Medical University Hospital, Taichung, 40447 Taiwan; 10 Department of Long-Term Care, College of Nursing, Asia University, Taichung, 413 Taiwan

**Keywords:** Pyogenic liver abscess (PLA), Sodium-glucose cotransporter-2 inhibitors (SGLT2i), Type 2 diabetes mellitus (T2DM), Propensity score, Cox proportional hazards models., Diseases, Endocrinology, Gastroenterology, Health care, Medical research, Risk factors

## Abstract

Pyogenic liver abscess (PLA) is a life-threatening infection rising in East Asia, especially among patients with type 2 diabetes. Although SGLT2 inhibitors improve glycemic control and offer extraglycemic benefits, their effect on PLA risk is unknown. Using Taiwan’s National Health Insurance Research Database, we conducted a nationwide retrospective cohort study of adults with T2DM. After 1:1 propensity score matching, 258,800 SGLT2i users and 258,800 non-users were included. The primary outcome was incident PLA. Incidence rates were calculated per 1,000 person-years, and adjusted hazard ratios (aHRs) with 95% confidence intervals (CIs) were estimated using multivariable Cox proportional hazards models. Additional analyses included subgroup analyses with interaction testing, a time-dependent Cox model, a competing-risks model, and a negative-control outcome analysis using fracture. During follow-up, 1,275 PLA events were identified. The incidence rate of PLA was 0.75 per 1,000 person-years in SGLT2i users and 0.83 per 1,000 person-years in non-users. In the primary multivariable Cox model, SGLT2i use was associated with a lower risk of PLA compared with nonuse (aHR, 0.88; 95% CI, 0.79–0.99). This inverse association was generally consistent across most subgroups. In the time-dependent analysis, SGLT2i use remained associated with a lower PLA risk (aHR, 0.72; 95% CI, 0.64–0.81). SGLT2i therapy was independently associated with reduced PLA risk in T2DM patients, particularly with prolonged exposure. These findings suggest an inverse association between SGLT2i use and the risk of pyogenic liver abscess in patients with T2DM.

## Introduction

Pyogenic liver abscess (PLA) is a potentially fatal intrahepatic infection that often necessitates advanced invasive interventions such as percutaneous drainage or surgery^1,2^. Population-based studies from Canada, Denmark, and the United States report PLA incidence rates of approximately 1.1 to 3.6 per 100,000 population, with mortality risk heightened in patients with liver cirrhosis, end-stage renal disease, or malignancy^3,4^. In Taiwan, the annual PLA incidence increased markedly, from 11.15 to 17.59 per 100,000 population between 1996 and 2004, underscoring significant regional epidemiologic differences^5^. Established risk factors include diabetes mellitus, liver cirrhosis, malignancy, and renal disease, and retrospective data indicate that Klebsiella pneumoniae PLA patients exhibit higher prevalences of diabetes, hypertension, and fatty liver disease^4–6^. Type 2 diabetes mellitus (T2DM) further predisposes individuals to PLA through mechanisms such as chronic hyperglycemia-induced immune dysfunction and hepatic steatosis^7,8^. Sodium-glucose cotransporter-2 inhibitors (SGLT2i) are a novel class of oral antihyperglycemic agents that lower blood glucose by promoting urinary glucose excretion^9,10^. Beyond glycemic control, SGLT2i confer renal protection, foster weight reduction, lower blood pressure, and improve non-alcoholic fatty liver disease. However, they have been associated with an increased risk of genitourinary infections^11–14^. To date, no study has evaluated whether SGLT2i use influences the risk of PLA in patients with T2DM^14,15^. Accordingly, we conducted a nationwide, propensity score-matched cohort study to compare PLA incidence rates between SGLT2i users and non-users with T2DM and to identify factors associated with PLA risk in this population.

## Materials and methods

### Data source

The National Health Insurance Research Database (NHIRD) was established in 1998, and Taiwan’s universal health coverage has currently achieved a participation rate of over 99%. The database has become a leading source of empirical data in healthcare. The NHIRD covers the period from 2009 to 2020, and we utilized data from 2016 to 2019. The NHIRD comprises comprehensive medical claims records for both outpatient and inpatient services, encompassing demographic information, diagnosis codes, medical interventions, and prescription data, including medication details, quantities, and dosages for insured individuals. To ensure individual privacy, all data undergo encryption and de-identification processes. The diagnoses in the database are based on the International Classification of Diseases, 9th Revision and 10th Revision, Clinical Modification (ICD-9-CM & ICD-10-CM, respectively).

### Study population

In this cohort study, we included patients with diabetes mellitus (DM) (ICD-9-CM: 250.0, 250.2; ICD-10-CM: E11). The exposed group consisted of patients who had used SGLT2 inhibitors (SGLT2i) for at least 90 days, while the control group comprised patients who had never used SGLT2i. The index date for the case group was defined as the first day of SGLT2i use, whereas the index date for the control group was randomly selected from the study period. Individuals under 20 years of age, with a history of pyogenic liver abscess before the index date, and those with missing baseline information were excluded. Propensity score matching was performed at a 1:1 ratio to match SGLT2i users and non-users based on gender, age, Insurance fee, urbanization, comorbidities, and medications.

### Primary outcome and relevant variables

The primary outcome is Pyogenic Liver Abscess (ICD-9-CM: 572.0; ICD-10-CM: K75.0). Related comorbidities include Alcoholism(ICD-9-CM: 291, 303, 305.0, 571.0–571.3.0.3; ICD-10-CM: V11.3 V79.1), Biliary stone(ICD-9-CM: 574; ICD-10-CM: K75.0), Chronic kidney disease (CKD) (ICD-9-CM: 585, 586, 588.8, 588.9, 593.9; ICD-10-CM: N18, N19, N25.8, N25.9), Chronic liver diseases (CLD) (ICD-9-CM: 571.0–571.3.0.3, 571.5, 571.6; ICD-10-CM: K70, K73, K74, K75.4), Hepatitis B virus (HBV) (ICD-9-CM: 070.2, 070.3, V02.61; ICD-10-CM: B16.0, B16.1, B16.2, B16.9, B18.0, B18.1, B19.10, B19.11, Z22.51), Hepatitis C virus (HCV) (ICD-9-CM: 070.41, 070.44, 070.51, 070.54, 070.70, 070.71, V02.62; ICD-10-CM: B17.10, B17.11, B19.20, B19.21, B18.2, Z22.52), and Liver transplantation. In terms of medications, we considered Metformin, Sulfonylureas, Glinides, alpha-glucosidase inhibitors (AGIs), Thiazolidinediones (TZDs), Dipeptidyl peptidase-4 inhibitors (DPP-4), Glucagon-like peptide-1 receptor agonists (GLP-1ra), and Insulin.

### Statistical analysis

We performed the analysis using SAS software version 9.4 (SAS Institute Inc., Cary, NC) and provided the mean and standard deviation for continuous variables, along with the count and percentage for categorical variables. Differences between the two groups were assessed using Student’s T-test and the chi-square test. Univariable and multivariable Cox proportional-hazards models were used to estimate risk ratios for each variable. Cumulative incidence probability curves for events were plotted using the Kaplan-Meier method, and the log-rank test was used for examination. The significance level for all tests was set at two-tailed *p* < 0.05.

We additionally performed subgroup analyses stratified by demographic characteristics, comorbidities, and concurrent antidiabetic medications, and tested interactions between SGLT2i use and subgroup variables. To address potential immortal-time bias, a time-dependent Cox proportional hazards model was further applied. Because death may preclude the occurrence of pyogenic liver abscess, a competing-risk analysis was also performed. In addition, fracture was analyzed as a negative control outcome to examine the possibility of residual confounding.

## Results

After 1:1 propensity score matching, 258,800 patients were included in each group. The distributions of sex, age, insurance fee, urbanization level, comorbidities, and concurrent antidiabetic medications were well balanced between the non-SGLT2i and SGLT2i groups, with all standardized mean differences below 0.1, indicating good covariate balance. The mean age was similar between the two groups (59.49 vs. 59.48 years). The proportions of women were also comparable (42.36% vs. 42.86%). In addition, the prevalence of major comorbidities, including CKD, CLD, hepatitis B or C virus infection, and heart failure, were similar between groups. The use of concurrent glucose-lowering medications, including metformin, Dipeptidyl peptidase-4 (DPP-4) inhibitors, Thiazolidinediones (TZDs), Glucagon-like peptide-1 (GLP-1) receptor agonists, and insulin, was likewise balanced after matching. Follow-up duration remained broadly comparable between groups, although the mean follow-up time was slightly longer in the SGLT2i group (3.29 vs. 2.96 years). The mean time from diabetes diagnosis to the index date and overall diabetes duration were also similar between groups.

**Table 1:** Baseline characteristics of T2DM patients with and without SGLT2i.

Covariates		Non-SGLT2i		SGLT2i		
		(*N* = 258800)		(*N* = 258800)		SMD
		n	%	n	%	
Sex	Female	109,623	42.36	110,932	42.86	0.01
	Male	149,177	57.64	147,868	57.14	0.01
Age	20–44	30,848	11.92	30,153	11.65	0.008
	45–69	178,565	69	178,875	69.12	0.003
	≥ 70	49,387	19.08	49,772	19.23	0.004
	Mean, (SD)	59.49	(12.14)	59.48	(12.05)	< 0.001
Insurance fee	Low	55,878	21.59	55,413	21.41	0.004
	medium	135,801	52.47	136,231	52.64	0.003
	high	67,121	25.94	67,156	25.95	< 0.001
Urbanization level	Low	22,956	8.87	23,114	8.93	0.002
	Median	101,650	39.28	101,880	39.37	0.002
	High	134,194	51.85	133,806	51.7	0.003
Comorbidites	Alcoholism	11,335	4.38	11,333	4.38	< 0.001
	Biliary stone	19,976	7.72	20,379	7.87	0.006
	CKD	42,163	16.29	41,322	15.97	0.009
	CLD	35,783	13.83	36,249	14.01	0.005
	HBV, HCV	29,195	11.28	29,399	11.36	0.002
	LT	190	0.07	199	0.08	0.001
	Heart failure	28,229	10.91	27,924	10.79	0.004
Medication	Metformin	251,596	97.22	252,131	97.42	0.013
	Sulfonylurea	969	0.37	967	0.37	< 0.001
	Glinides	18,885	7.3	18,806	7.27	0.001
	AGI	103,167	39.86	102,672	39.67	0.004
	TZD	110,050	42.52	108,819	42.05	0.01
	DPP4	186,767	72.17	186,252	71.97	0.004
	GLP1ra	6766	2.61	6482	2.5	0.007
	Insulin	115,155	44.5	114,130	44.1	0.008
Follow-up years, mean (SD)		2.96	(1.46)	3.29	(1.37)	0.235
Years from DM to the index date, mean (SD)		2.11	(1.40)	2.08	(1.38)	0.019
DM duration, mean (SD)		8.20	(5.76)	8.23	(5.58)	0.004

SMD: standard mean difference; SGLT2i: SGLT2 inhibitors, Insurance fee, low (< 670 USD), medium (670-1,350 USD), high (> 1,350 USD); CKD: Chronic kidney disease; CLD: Chronic liver diseases; HBV: Hepatitis B virus; HCV: Hepatitis C virus; AGI: Alpha-glucosidase inhibitor; TZD: Thiazolidinediones; DPP4: Dipeptidyl peptidase-4 inhibitor; GLP1ra: Glucagon-like peptide-1.

During follow-up, 1,275 cases of PLA were identified. The incidence rate of PLA was 0.83 per 1,000 person-years in the non-SGLT2i group and 0.75 per 1,000 person-years in the SGLT2i group. In the multivariable Cox proportional hazards model, SGLT2i use was associated with a significantly lower risk of PLA compared with nonuse (adjusted hazard ratio [aHR], 0.88; 95% CI, 0.79–0.99). Male sex was associated with a higher risk of PLA than female sex (aHR, 1.30; 95% CI, 1.16–1.47). Among comorbidities, alcoholism, biliary stone disease, chronic liver disease, and hepatitis B or C virus infection were associated with increased PLA risk. Regarding concurrent medications, glinide use and insulin use were associated with higher PLA risk, whereas most other antidiabetic medications were not significantly associated with the outcome. (Tables 1, 2).

**Table 2:** Incidence rate and hazard ratio of Pyogenic Liver Abscess (*n* = 1,275) in T2DM patients with and without SGLT2i by Cox model.

Covariates		Event	PY	IR	aHR	95% CI
SGLT2i	No	637	765,959	0.83	1.00	
	Yes	638	852,062	0.75	0.88	0.79, 0.99
Sex	Female	482	702,160	0.69	1.00	
	Male	793	915,861	0.87	1.3	1.16, 1.47
Age	20–44	104	194,142	0.54	1.00	
	45–69	847	1,143,952	0.74	0.87	0.66, 1.15
	≥ 70	324	279,928	1.16	0.91	0.60, 1.37
Insurance fee	Low	309	338,315	0.91	1.00	
	medium	659	853,680	0.77	0.92	0.80, 1.05
	high	307	426,026	0.72	0.92	0.79, 1.08
Urbanization	Low	110	142,567	0.77	1.00	
	Median	523	635,191	0.82	1.09	0.89, 1.34
	High	642	840,264	0.76	1.05	0.86, 1.29
Comorbidites	Alcoholism	109	67,092	1.63	1.66	1.32, 2.08
	Biliary stone	193	126,850	1.52	1.69	1.44, 1.97
	CKD	256	238,945	1.07	1.14	0.98, 1.31
	CLD	253	210,429	1.20	1.37	1.16, 1.61
	HBV, HCV	195	178,618	1.09	1.22	1.04, 1.44
	LT	4	1176	3.40	2.19	0.81, 5.89
	Heart failure	175	166,474	1.05	1.01	0.85, 1.19
Medication	Metformin	1250	1,580,812	0.79	0.91	0.60, 1.37
	Sulfonylurea	9	6507	1.38	1.45	0.75, 2.80
	Glinides	130	107,700	1.21	1.36	1.13, 1.63
	AGI	602	662,351	0.91	0.99	0.87, 1.12
	TZD	652	700,445	0.93	1.14	1.00, 1.29
	DPP4	984	1,174,353	0.84	1.06	0.92, 1.22
	GLP1ra	29	39,717	0.73	0.87	0.60, 1.26
	Insulin	748	711,752	1.05	1.56	1.38, 1.77

Event: number of patients with pyogenic liver abscess; IR: Incidence rate, per 1000 persons/years; aHR: adjusted HR (adjusted for Sex, Insurance fee, Urbanization level, comorbidities, and medication); CI: confidence interval; LT: liver transplantation.

In subgroup analyses, the association between SGLT2i use and lower PLA risk was generally consistent across most strata. The adjusted hazard ratio was 0.88 (95% CI, 0.74–1.06) in women and 0.89 (95% CI, 0.77–1.02) in men, with no significant interaction by sex (P for interaction = 0.84). Similarly, no significant interaction was observed across age groups, insurance fee categories, urbanization levels, alcoholism, biliary stone disease, chronic kidney disease, chronic liver disease, hepatitis B or C virus infection, liver transplantation status, or heart failure. A lower PLA risk associated with SGLT2i use was observed in several clinically relevant strata, including patients with chronic liver disease (aHR, 0.74; 95% CI, 0.58–0.95), those without hepatitis B or C virus infection (aHR, 0.88; 95% CI, 0.78–0.99), metformin users (aHR, 0.87; 95% CI, 0.78–0.98), and patients not receiving sulfonylureas (aHR, 0.88; 95% CI, 0.79–0.99). Significant interactions were observed for glinide use (P for interaction = 0.02) and insulin use (P for interaction = 0.02). In these strata, the inverse association between SGLT2i use and PLA appeared more evident among patients receiving glinides (aHR, 0.61; 95% CI, 0.43–0.87) and those receiving insulin (aHR, 0.80; 95% CI, 0.70–0.93). Overall, these findings suggest that the association between SGLT2i use and PLA risk was broadly consistent, although potential heterogeneity may exist by concurrent antidiabetic therapies. Data for the liver transplantation subgroup were limited, and several cells were suppressed because of small counts to preserve patient confidentiality. (Table 3).

**Table 3:** Incidence and hazard ratios of Pyogenic Liver Abscess in T2DM patients with and without SGLT2i by age, gender, and comorbidity using Cox model.

Covaiates		non-SGLT2i			SGLT2i			aHR (95% CI)	*P* for interaction
		Event	PY	Rate ^†^	Event	PY	Rate ^†^		
Sex	female	241	330,204	0.73	241	371,957	0.65	0.88 (0.74, 1.06)	0.84
	male	396	435,756	0.91	397	480,106	0.83	0.89 (0.77, 1.02)	
Age	20 − 64	52	91,103	0.57	52	103,039	0.51	0.86 (0.58, 1.27)	0.88
	65 − 74	423	541,162	0.78	424	602,790	0.70	0.87 (0.76, 1.00)	
	≥ 75	162	133,694	1.21	162	146,233	1.11	0.93 (0.74, 1.15)	
Insurance fee	Low	160	158,419	1.01	149	179,897	0.83	0.82 (0.65, 1.02)	0.61
	medium	322	405,343	0.79	337	448,337	0.75	0.93 (0.80, 1.08)	
	high	155	202,197	0.77	152	223,829	0.68	0.89 (0.71, 1.11)	
Urbanization	Low	57	67,847	0.84	53	74,720	0.71	0.84 (0.58, 1.23)	0.75
	Median	254	300,655	0.85	269	334,535	0.80	0.93 (0.79, 1.11)	
	High	326	397,457	0.82	316	442,807	0.71	0.85 (0.73, 0.99)	
Alcoholism	No	578	735,290	0.79	588	815,639	0.72	0.90 (0.81, 1.01)	0.20
	Yes	59	30,669	1.92	50	36,423	1.37	0.73 (0.50, 1.06)	
Biliary stone	No	532	706,928	0.75	550	784,244	0.70	0.91 (0.81, 1.03)	0.12
	Yes	105	59,032	1.78	88	67,818	1.30	0.75 (0.57, 1.00)	
CKD	No	497	651,690	0.76	522	727,386	0.72	0.90 (0.80, 1.02)	0.12
	Yes	140	114,269	1.23	116	124,676	0.93	0.80 (0.62, 1.03)	
CLD	No	500	668,026	0.75	522	739,566	0.71	0.93 (0.82, 1.05)	0.08
	Yes	137	97,933	1.40	116	112,496	1.03	0.74 (0.58, 0.95)	
HBV, HCV	No	541	682,665	0.79	539	756,739	0.71	0.88 (0.78, 0.99)	0.99
	Yes	96	83,295	1.15	99	95,323	1.04	0.90 (0.68, 1.19)	
LT	No	NA	NA	NA	NA	NA	NA	0.88 (0.79, 0.98)	0.39
	Yes	NA	NA	NA	NA	NA	NA	NA	
Heart failure	No	545	689,119	0.79	555	762,428	0.73	0.89 (0.79, 1.01)	0.27
	Yes	92	76,840	1.20	83	89,634	0.93	0.79 (0.59, 1.08)	
Metformin	No	10	19,312	0.52	15	17,897	0.84	1.40 (0.59, 3.32)	0.14
	Yes	627	746,647	0.84	623	834,165	0.75	0.87 (0.78, 0.98)	
Sulfonylurea	No	632	762,990	0.83	634	848,525	0.75	0.88 (0.79, 0.99)	0.66
	Yes	5	2970	1.68	4	3538	1.13	0.61 (0.16, 2.38)	
Glinides	No	560	715,558	0.78	585	794,764	0.74	0.92 (0.82, 1.03)	0.02
	Yes	77	50,402	1.53	53	57,298	0.93	0.61 (0.43, 0.87)	
AGI	No	330	458,578	0.72	343	497,092	0.69	0.92 (0.79, 1.08)	0.20
	Yes	307	307,381	1.00	295	354,970	0.83	0.84 (0.72, 0.99)	
TZD	No	315	439,617	0.72	308	477,959	0.64	0.87 (0.74, 1.02)	0.95
	Yes	322	326,342	0.99	330	374,103	0.88	0.90 (0.77, 1.05)	
DPP4	No	139	221,375	0.63	152	222,294	0.68	1.06 (0.84, 1.34)	0.05
	Yes	498	544,584	0.91	486	629,769	0.77	0.84 (0.74, 0.95)	
GLP1ra	No	623	747,617	0.83	623	830,688	0.75	0.88 (0.79, 0.99)	0.94
	Yes	14	18,342	0.76	15	21,375	0.70	0.87 (0.41, 1.85)	
Insulin	No	250	437,509	0.57	277	468,761	0.59	1.00 (0.84, 1.19)	0.02
	Yes	387	328,450	1.18	361	383,301	0.94	0.80 (0.70, 0.93)	

Event: number of patients with pyogenic liver abscess; IR: Incidence rate, per 1000 persons/years; aHR: adjusted HR (adjusted for Sex, Insurance fee, Urbanization level, comorbidities, and medication); CI: confidence interval; LT: liver transplantation; NA: not available, data were suppressed due to small cell counts to protect patient confidentiality.

Tables 4, 5, 6 shows the time-dependent analysis; a time-dependent model was further applied. In this analysis, SGLT2i use remained associated with a lower risk of PLA compared with nonuse (aHR, 0.72; 95% CI, 0.64–0.81). The inverse association was observed in both women (aHR, 0.79; 95% CI, 0.65–0.95) and men (aHR, 0.69; 95% CI, 0.60–0.80), and across most age groups, particularly among patients aged 20–64 years and 65–74 years. Similar inverse associations were also observed across multiple clinical subgroups, including patients with alcoholism, biliary stone disease, chronic kidney disease, chronic liver disease, hepatitis C virus infection, heart failure, and users of metformin, glinides, AGIs, TZDs, DPP-4 inhibitors, and insulin. Although some subgroup-specific estimates were imprecise due to limited event counts, the overall findings of the time-dependent analysis were directionally consistent with the primary analysis and continued to support an inverse association between SGLT2i use and PLA risk.

**Table 4:** Incidence and hazard ratios of Pyogenic Liver Abscess in T2DM patients with and without SGLT2i by age, gender, and comorbidity using time dependent model.

Covariates		SGLT2i compared to non-SGLT2i
		aHR (95% CI)
SGLT2i (No vs. Yes)		0.72 (0.64, 0.81)
Sex	Female	0.79 (0.65, 0.95)
	Male	0.69 (0.60, 0.80)
Age	20 − 64	0.53 (0.35, 0.83)
	65 − 74	0.71 (0.62, 0.82)
	≥ 75	0.82 (0.66, 1.04)
Insurance fee	Low	0.58 (0.45, 0.74)
	medium	0.80 (0.68, 0.93)
	high	0.74 (0.58, 0.93)
Urbanization	Low	0.96 (0.65, 1.41)
	Median	0.69 (0.58, 0.83)
	High	0.71 (0.60, 0.83)
Alcoholism	No	0.74 (0.65, 0.83)
	Yes	0.59 (0.39, 0.89)
Biliary stone	No	0.73 (0.65, 0.83)
	Yes	0.66 (0.49, 0.89)
CKD	No	0.73 (0.64, 0.83)
	Yes	0.66 (0.51, 0.87)
CLD	No	0.77 (0.68, 0.87)
	Yes	0.56 (0.42, 0.73)
HCV	No	0.71 (0.53, 0.95)
	Yes	0.74 (0.55, 0.99)
LT	No	0.72 (0.64, 0.81)
	Yes	0.18 (0.00, 158.3)
Heart failure	No	0.74 (0.66, 0.84)
	Yes	0.58 (0.41, 0.82)
Metformin	No	1.09 (0.47, 2.50)
	Yes	0.71 (0.63, 0.80)
Sulfonylurea	No	0.72 (0.64, 0.81)
	Yes	0.39 (0.08, 1.89)
Glinides	No	0.75 (0.66, 0.84)
	Yes	0.52 (0.35, 0.77)
AGI	No	0.76 (0.65, 0.89)
	Yes	0.68 (0.57, 0.81)
TZD	No	0.69 (0.59, 0.82)
	Yes	0.75 (0.64, 0.88)
DPP4	No	0.77 (0.61, 0.99)
	Yes	0.71 (0.62, 0.81)
GLP1ra	No	0.72 (0.64, 0.81)
	Yes	0.78 (0.36, 1.70)
Insulin	No	0.82 (0.68, 0.98)
	Yes	0.65 (0.56, 0.76)

IR: Incidence rate, per 1000 persons/years; aHR: adjusted HR (adjusted for Sex, Insurance fee, Urbanization level, comorbidities, and medication); CI: confidence interval; LT: liver transplantation.

Because death may preclude the occurrence of PLA, a competing-risk model was additionally performed. In this analysis, SGLT2i use remained associated with a lower subdistribution hazard of PLA than nonuse (adjusted subhazard ratio [aSHR], 0.88; 95% CI, 0.79–0.97). The magnitude and direction of the association were similar to those observed in the primary Cox model, suggesting that the main findings were robust after accounting for death as a competing event.

**Table 5:** Incidence rate and subhazard ratio of Pyogenic Liver Abscess (*n* = 1,712) in T2DM patients with and without SGLT2i by competing risk model.

Covariates	cSHR	95% CI	aSHR	95% CI
SGLT2i				
No	1.00		1.00	
Yes	0.87	0.79, 0.97	0.88	0.79, 0.97

cSHR: crude subhazard ratio; aSHR: adjusted subhazard ratio (adjusted for Sex, Insurance fee, Urbanization level, comorbidities, and medication); CI: confidence interval.

To further examine the possibility of residual confounding, fracture was used as a negative control outcome. The fracture incidence rate was 23.9 per 1,000 person-years in the non-SGLT2i group and 24.7 per 1,000 person-years in the SGLT2i group. In the adjusted analysis, SGLT2i use was not significantly associated with fracture risk (aHR, 1.04; 95% CI, 0.94–1.14). This null finding for the negative control outcome provides additional support that the observed association between SGLT2i use and PLA was less likely to be entirely explained by systematic bias.

**Table 6:** Incidence rate and hazard ratio of fractures (Negative Control Outcome) in T2DM patients with and without SGLT2i.

Covariates		Event	PY	IR	aHR	95% CI
SGLT2i	No	806	33,695	23.9	1.00	(reference)
	Yes	962	38,882	24.7	1.04	(0.94, 1.14)

Event: number of patients with pyogenic liver abscess; IR: Incidence rate, per 1000 persons/years; aHR: adjusted HR (adjusted for Sex, Insurance fee, Urbanization level, comorbidities, and medication); CI: confidence interval.

**Fig. 1:**
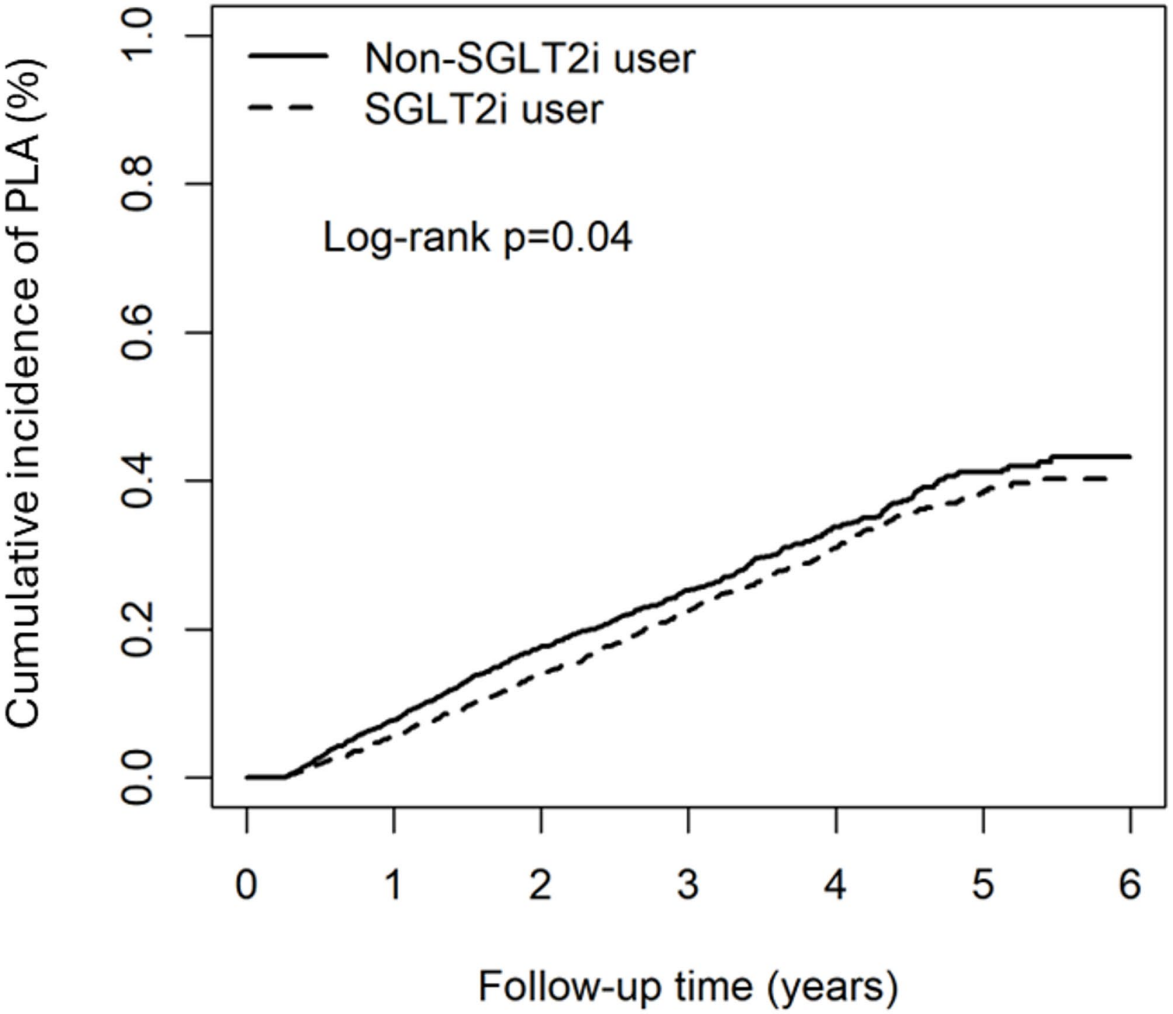
Kaplan–Meier cumulative incidence curves for pyogenic liver abscess in patients with type 2 diabetes mellitus according to SGLT2 inhibitor use.

The cumulative incidence of PLA was compared between SGLT2i users and non-SGLT2i users during follow-up. The dashed line represents SGLT2i users, and the solid line represents non-SGLT2i users. The cumulative incidence of PLA was lower in the SGLT2i group than in the non-SGLT2i group, and the difference between the two groups was significant by the log-rank test (*p* = 0.04). (Fig. 1)

## Discussion

In the primary multivariable analysis, SGLT2i use was associated with a lower risk of PLA, whereas male sex, alcoholism, biliary stone disease, chronic liver disease, hepatitis B or C virus infection, glinide use, and insulin use were associated with a higher risk.

One retrospective study showed that diabetic patients have a higher risk and incidence rate of liver abscess. In DM patients, there is a higher incidence rate in DM diagnosed within 2 years, males, history of gallstone and cholecystitis, and glyburide users, and a lower incidence rate in DM patients using α-glucosidase inhibitors. However, this study didn’t analyze the effect of SGLT2i use^16^. Our study compares the prevalence of PLA between SGLT2i users and non-users. In our analysis, SGLT2i use was associated with a lower observed risk of PLA, and this association appeared somewhat stronger in males than in females. A similar association was observed among sulfonylurea users, glinide users, insulin users, and patients with longer SGLT2i exposure, but not among those with biliary stone-related disease.

Obesity and metabolic syndrome may impair immune response^17^. There’s a higher prevalence of liver abscess in diabetic patients, especially those with poor glycemic control^17^, and diabetic patients may also have a high risk of septic metastatic lesions, ex, endophthalmitis, uveitis, and pulmonary abscess^18^. In one retrospective study, diabetes, hypertension, and fatty liver are risk factors for pyogenic liver abscess^19^. Among anti-diabetic drugs, SGLT2i have been reported to improve glycemic control and are also associated with reductions in body weight and modest decreases in blood pressure.

The gut-liver axis affects bacterial translocation, inflammation, and infection^20^. Klebsiella pneumoniae is one of the most common pathogens in PLA, and a study showed that K. pneumoniae originates from the gastrointestinal flora of patients and healthy individuals in Taiwan, suggesting that leakage of K. pneumoniae from a patient’s gastrointestinal tract into the portal vein may lead to liver abscess^21^. In drug effect, patients have a lower risk of recurrence of pyogenic liver abscess if using aspirin, and the use of metformin in diabetic patients for more than 90 days reduces the risk of PLA^22,23^. The use of aspirin or metformin may alter the gut microbiota, but whether this affects PLA remains to be investigated^24,25^. In the SGLT2i model, dapagliflozin alters gut flora, and the combination of dapagliflozin and metformin exhibits a complementary effect^26^. The impact of SGLT2i on gut flora requires further study.

A nationwide study in Denmark revealed that the risk and case fatality rate of pyogenic liver abscess are increased in patients with liver cirrhosis^27^. Obesity and fatty liver contribute to liver cirrhosis. Beyond their metabolic effects, SGLT2i have been reported to be associated with lower inflammatory and oxidative stress markers and with improvement in fatty liver-related measures in some studies^28^. Some studies have reported that SGLT2i use was associated with lower liver fat content on imaging^29,30^ and with potentially favorable hepatic outcomes^29^. In one study, the use of SGLT2i Empagliflozin in diabetic patients decreased the inflammatory profile and improved the antioxidative response in leukocytes^30^. One sub-analysis of a randomized controlled study reported that ipragliflozin was associated with improvements in hepatic steatosis and liver fibrosis^31^. Additionally, a retrospective study revealed that compared with DPP4i, SGLT2i may lower the risk of mortality in patients with cirrhosis^31^.

Despite its nationwide scope and reduced bias, limitations persist. We used propensity score matching on demographics and comorbidities to ensure comparability between SGLT2i users and non-users; however, this approach may have yielded a control group whose underlying risk profile did not fully mirror that of the source population, potentially underestimating PLA risk. As the PLA evolves, our mean follow-up of approximately two years (dictated by the enrollment window) may have missed incident cases occurring outside the study period, further biasing risk estimates downward. Although most participants resided in high- or medium-urbanization areas, raising the possibility of surveillance bias due to unequal healthcare access, 99% of residents are covered by Taiwan’s National Health Insurance, which provides free services even in remote regions, helping to minimize this concern^32,33^. Finally, the NHIRD does not capture key hematologic and biochemical parameters (e.g., leukocyte counts, ESR/CRP, transaminases, bilirubin, alkaline phosphatase), so we were unable to adjust for these covariates. Furthermore, detailed information on insulin subtypes, treatment adherence, and the clinical reasons for SGLT2i discontinuation or reinitiation was unavailable in the claims database. Finally, although we performed time-dependent and competing-risk analyses, residual confounding and time-related bias cannot be completely excluded.

In conclusion, an inverse association was observed between SGLT2i use and PLA risk in patients with T2DM in this nationwide retrospective cohort study. Although SGLT2i may increase the risk of urinary tract infection, our study adds to the emerging evidence regarding a possible association between SGLT2i use and lower PLA risk, although causal inference remains limited. The role of SGLT2i in the gut-liver axis remains unclear, and further studies are necessary to confirm this.

## Data Availability

Information is accessible through the National Health Insurance Research Database (NHIRD) provided by the Taiwan National Health Insurance (NHI) Administration. However, due to legal constraints stipulated by the Taiwan government under the Personal Information Protection Act, the data cannot be made public. To obtain data, researchers may submit formal requests to the NHIRD via its website (https://dep.mohw.gov.tw/DOS/lp-2506-113.html).The datasets generated and/or analyzed during the current study are not publicly available due to these legal restrictions. Still, they are available from the corresponding author, Dr. Kuang-Hsi Chang (email: kuanghsichang@gmail.com), upon reasonable request.
